# The application of dental mesenchymal stem cells (DMSCs) in the repair of oral and maxillofacial defects

**DOI:** 10.3389/fbioe.2025.1677422

**Published:** 2025-10-03

**Authors:** Yiqi Chen, Xikun Ma, Huaqing Mai, Pengyu Lai, Renlong Huang, Adili Alimujiang

**Affiliations:** ^1^ School of Stomatology, Jinan University, Guangzhou, China; ^2^ State Key Laboratory of Oral & Maxillofacial Reconstruction and Regeneration, Key Laboratory of Oral Biomedicine Ministry of Education, Hubei Key Laboratory of Stomatology, School & Hospital of Stomatology, Wuhan University, Wuhan, China

**Keywords:** dental mesenchymal stem cells, dental pulp stem cells, periodontalligament stem cells, deciduous tooth pulp stem cells, gingival mesenchymal stemcells, apical papilla stem cells, dental follicle precursor cells, tissue regeneration

## Abstract

Dental mesenchymal stem cells (DMSCs) are important seed cells for oral and maxillofacial tissue regeneration due to their wide availability, multi-directional differentiation potential, strong proliferation ability, maintenance of stemness after multiple proliferations, and immunomodulatory capacity. DMSCs have a wide range of sources, and different subtypes have different properties. They can promote tissue regeneration through direct differentiation into tissue cells, improvement of the local regeneration microenvironment through immunomodulatory functions, and release of various bioactive components. The article reviews the classification of DMSCs, their performance comparisons in recent years, methods of promoting tissue regeneration, and their applications in maxillofacial bone and soft tissue. Furthermore, the review explores DMSCs’ excellent abilities to promote bone tissue repair when supported by various materials and repair maxillofacial soft tissue defects by promoting the regeneration of special structures of teeth such as dentin, cementum, and dental pulp vessels and nerves.

## 1 Introduction

Oral and maxillofacial defects are often caused by trauma, tumors or congenital diseases. For a long time, traditional repair methods have faced many insurmountable bottlenecks in restoring the function of the oral and maxillofacial region and meeting aesthetic needs. However, in recent years, with the vigorous development of the frontier field of tissue regeneration medicine, a brand-new exploration direction has been opened up for the repair of oral and maxillofacial defects. Among many innovative approaches, dental mesenchymal stem cells (DMSCs) have stood out due to their wide availability, multi-directional differentiation potential, and low immunogenicity, becoming one of the most notable hotspots in current oral medicine research.

Mesenchymal stem cells (MSCs) are a type of non-hematopoietic adult stem cells. They can be isolated from various tissues and organs, including bone marrow, skin, adipose tissue, and umbilical cord. These cells possess the potential for both proliferation and differentiation (Li et al., 2023). DMSCs are a type of adult stem cells among mesenchymal stem cells and can be isolated from dental pulp, periodontal ligament, apical papilla, gingiva, dental follicle and human deciduous teeth. Due to their multi-directional differentiation potential, strong proliferation ability, maintenance of cell stemness after multiple proliferations, and immunomodulatory capacity, they have become an important seed cell in the clinical application of tissue regeneration.

## 2 The classification of DMSCs

**FIGURE 1:**
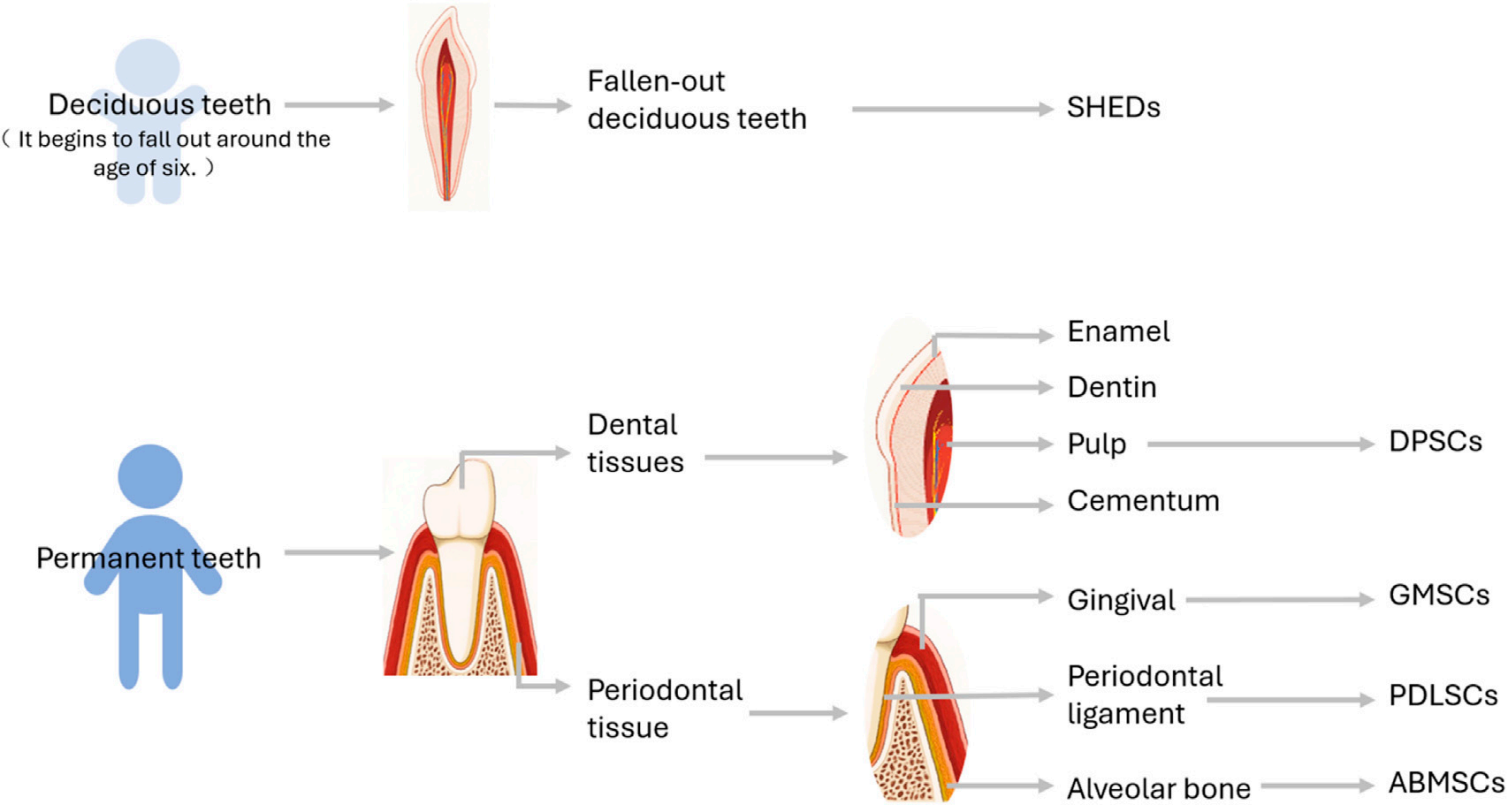
Schematic diagram of the Origin of DMSCs. SHEDs: Stem cells populations from human exfoliated deciduous teeth, DPSCs, Dental pulp stem cells; GMSCs, Gingival mesenchymal stem cells; PDLSCs, Periodontal ligament stem cells; ABMSCs, Alveolar bone mesenchymal stem cells.

**FIGURE 2:**
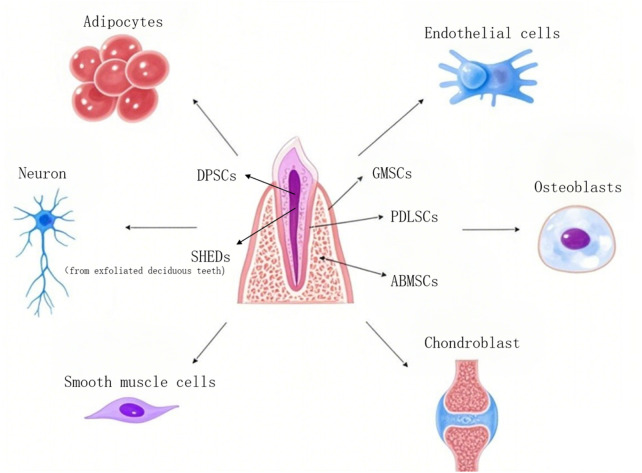
Partial sources and differentiation of dental mesenchymal stem cells. DPSCs, Dental pulp stem cells; SHEDs, Stem cells populations from human exfoliated deciduous teeth; GMSCs, Gingival mesenchymal stem cells; PDLSCs, Periodontal ligament stem cells; ABMSCs, Alveolar bone mesenchymal stem cells.

DMSCs are characterized by a diverse array of sources, and their properties vary notably among different subtypes. In the subsequent part, the sources of DMSCs are introduced in Figure 1. Additionally, several common types of DMSCs and their properties are expounded upon. Moreover, Figure 2 illustrates the sources and differentiation potential of DMSCs.

### 2.1 Dental pulp stem cells (DPSCs)

Dental pulp refers to the loose connective tissue within the pulp cavity surrounded by dentin, which has functions such as forming dentin, sensing pain, providing nutrition, and resisting bacterial invasion. Dental pulp stem cells are cells isolated from dental pulp with mesenchymal stem cell characteristics. In 2000, Gronthos et al. successfully isolated DPSCs (Gronthos et al., 2000). As the earliest isolated dental stem cells, they revealed that mesenchymal stem cells also exist in dental-related tissues besides bone marrow, adipose tissue, placenta, umbilical cord, etc. Compared with the extraction of bone marrow mesenchymal stem cells which requires anesthesia, DPSCs are easier to obtain and can be isolated from healthy first premolars, third molars, or other teeth extracted for orthodontic purposes, thus causing less ethical disputes (Hwan and Woo, 2022).

With the deepening of research, DPSCs can be traced back to neural crest cells, thus having the ability to differentiate into neurons and glial cells. Under certain environmental conditions, they can also produce capillary-like structures by synthesizing and secreting angiogenic regulators. Based on their potential for neural differentiation and angiogenesis, dental pulp stem cells have become the best seed cells for dental pulp regeneration.

### 2.2 Periodontal ligament stem cells (PDLSCs)

The periodontal ligament is a dense connective tissue connecting the alveolar bone and cementum. As a connection between the alveolar bone and cementum, it plays a crucial role in resisting and regulating the pressure on teeth during chewing. In 2004, Seo et al. successfully isolated periodontal ligament stem cells (Seo et al., 2004). *In vitro* experiments have shown that when periodontal tissue is damaged, periodontal ligament stem cells can migrate to the damaged area and repair it, revealing the possibility of periodontal tissue regeneration (Hwan and Woo, 2022).

### 2.3 Stem cells populations from human exfoliated deciduous teeth (SHEDs)

In 2003, Miura et al. discovered and isolated SHEDs (Miura et al., 2003). Due to the growth and development differences between deciduous teeth and permanent teeth, deciduous dental pulp stem cells exhibit different characteristics from permanent dental pulp stem cells, such as a strong proliferation tendency, higher mineralization ability, and unique osteoinductive ability. Therefore, they have become a new direction for the repair of dental and bone tissues in the future (Forough et al., 2023).

### 2.4 Gingival mesenchymal stem cells (GMSCs)

GMSCs were first proposed by Zhang et al., in 2009 (Zhang et al., 2009). The gingiva is a tough oral mucosa surrounding the cervical part of the tooth and the alveolar ridge, and it participates in oral mucosal immunity as a mucosal barrier. Studies have found that gingival mesenchymal stem cells have the potential for bone regeneration, neural regeneration, and the regeneration of muscle, cartilage, and synovial tissues, but their osteogenic potential is lower than that of other sources of dental mesenchymal stem cells (Gamilah et al., 2021).

### 2.5 Other DMSCs

In addition to the above-mentioned stem cells, there are also dental follicle stem cells (DFSCs) (also known as dental follicle progenitor cells, DFPCs), (Karamzadeh et al., 2017), alveolar bone mesenchymal stem cells (ABMSCs), (Mason et al., 2014), and stem cells from apical papilla (SCAPs), etc (Sonoyama et al., 2006).

## 3 Comparison of various cellular properties of DMSCs

The following table compares the proliferation ability, differentiation potential and immune regulation characteristics of several types of cells (Table 1).

**TABLE 1:** Comparison of various properties of DMSCs (Guanlin et al., 2021; Linglu et al., 2021; Lu et al., 2020; Sabbagh et al., 2020; Al-Qadhi et al., 2021; Deng et al., 2024; Kotova et al., 2021; Nowwarote et al., 2020; Abe et al., 2022).

Cellular type	Proliferation capacity	Differentiation potential	Immune regulation	Cell markers
DPSCs	The proliferation rate is similar to that of PDLSCs but lower than that of DFPCs. The proliferation capacity and osteoinductive ability are higher than those of DPSCs	Endothelial cells, osteoblasts, adipocytes, chondroprogenitor cells, hepatocytes and cardiomyocytes have neurogenic differentiation potential. Among them, the osteogenic differentiation ability is greater than that of DFPCs	It has immunomodulatory properties, can inhibit T cell proliferation, trigger M2 macrophage polarization, and suppress inflammation, etc.	Stro-1, CD49f, CD29, CD44、CD105, CD90
SHEDs	The proliferation rate is similar to that of PDLSCs but lower than that of DFPCs. The proliferation capacity and osteoinductive ability are higher than those of DPSCs	Osteocytes, chondrocytes, adipocytes, odontoblasts, endothelial cells and hepatocytes Unique dental tissue and osteoinductive ability* Can be used as a neuroprotective agent*	/	CD73, CD90, CD105 CD146
PDLSCs	/	The differentiation potential of osteocytes, endothelial cells, cardiomyocytes, islet-like cells and retinal ganglion-like cells It can form typical cementum and periodontal ligament-like structures	/	Stro-1, CD146, CD29, CD44, CD106
GMSCs	Greater than PDLSCs, and the degree of senescence after continuous passage *in vitro* culture is less than that of PDLSCs. Compared with DPSCs and PDLSCs, it has a unique ability to automatically differentiate into gingiva *in vivo*	Neurogenic differentiation, adipocytes, chondrocytes and endothelial cells	/	CD13, CD29, CD44, CD54, CD73, CD90, CD105, CD166, Stro-1

Unique dental tissue and bone induction ability*: Capable of differentiating into odontoblasts and forming dentin-like or pulp-like tissues, rather than complete dentin-pulp-like complexes. *In vivo*, it recruits host osteoblasts to induce new bone formation, unlike *in vitro* where it differentiates into osteoblasts.

Can act as a neuroprotective agent*: Promotes neural function recovery through paracrine action and inhibits the formation of glial scars after spinal cord contusion.

Unique ability to automatically differentiate into gingiva in vivo*: Transplantation forms connective-like tissue expressing collagen I, a capability not possessed by DPSCs, and PDLSCs.

DPSCs, Dental pulp stem cells; SHEDs, Stem cells populations from human exfoliated deciduous teeth; PDLSCs, Periodontal ligament stem cells; GMSCs, Gingival mesenchymal stem cells; DFPCs, Dental follicle progenitor cells.

## 4 The modalities by which dental mesenchymal stem cells facilitate tissue regeneration

Previous studies have shown that dental mesenchymal stem cells mainly promote tissue regeneration through three ways: (1)differentiation into tissue cells; (2) improvement of the local regeneration microenvironment through immunomodulatory functions; (3) promotion of tissue regeneration by releasing various bioactive components.

### 4.1 Differentiate into tissue cells

During the development of the tooth germ, the enamel organ composed of the ectodermal epithelium undergoes significant morphological changes. In the cap stage, an intermediate layer appears between the inner enamel epithelium and the stellate reticulum. In the late stage, acidic mucopolysaccharides and glycogen deposits can be observed in the cytoplasm of the intermediate layer cells, and they have high alkaline phosphatase activity. Subsequent studies have demonstrated that alkaline phosphatase is involved in the formation of enamel, dentin, and bone. Furthermore, its high enzymatic activity can serve as a marker for cells associated with the development of these tissues (Yu et al., 2015). Stem cell surface markers are often used to routinely identify the differentiation potential of pluripotent mesenchymal stem cells (i.e., to confirm the strength of the stemness of cells). CD51/CD140α and CD271 have been used to isolate and identify neural crest cell progenitors, while the STRO-1/CD146 combination has been used to isolate mesenchymal stem cells (MSCs) located in blood vessels (Mao et al., 2012). Since the success of craniofacial defect repair depends on the ability to separate specific subpopulations of DMSCs with strong differentiation ability into appropriate cell types, it is crucial to identify effective markers for isolating pluripotent DMSCs. The mRNA expression of dental source genes can indicate whether mesenchymal stem cells have differentiated into dental lineage tissue cells.

**FIGURE 3:**
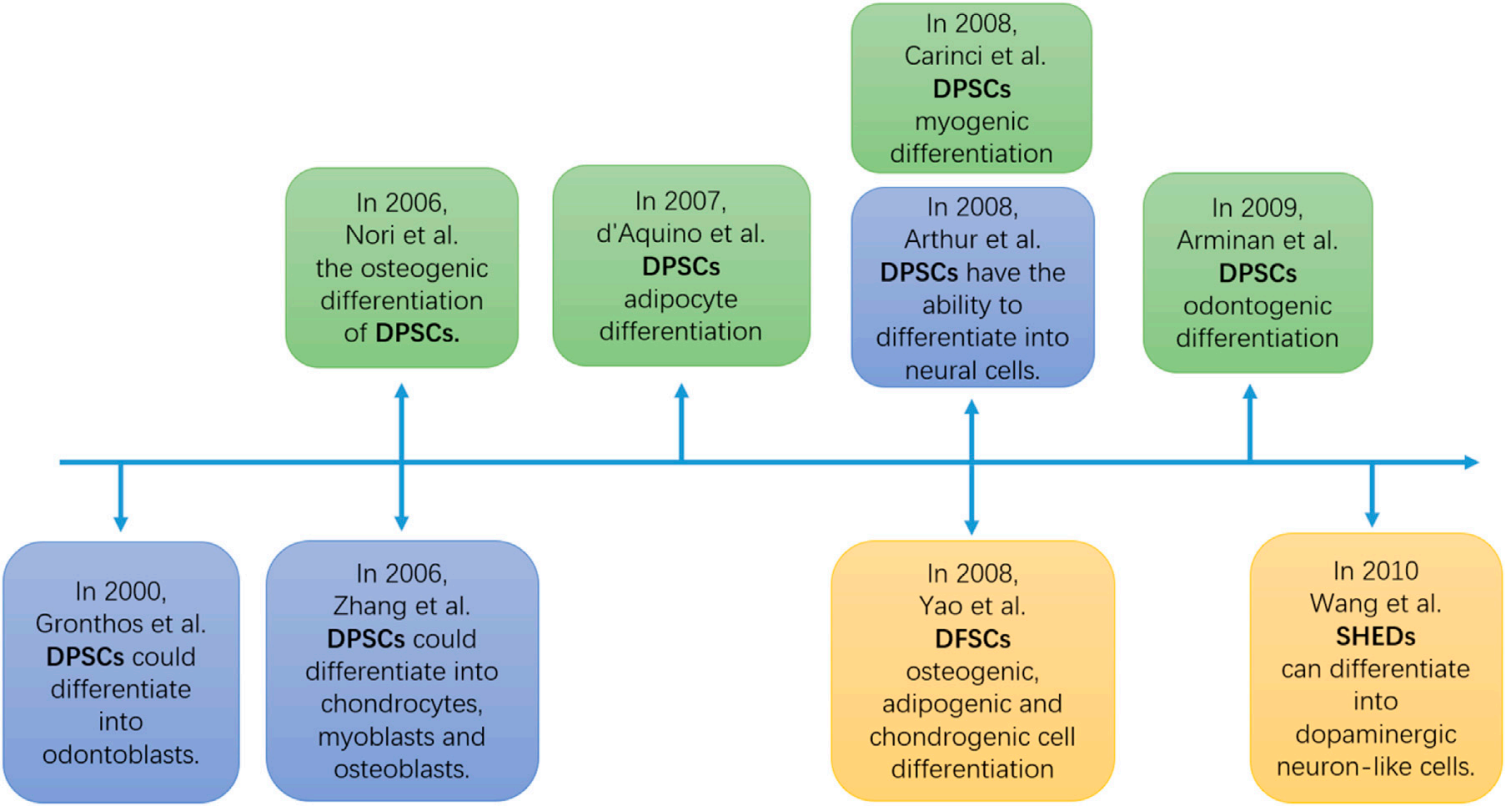
The timeline of the DMSCs differentiation potential experimen (Zhang et al., 2006; Arthur et al., 2008; Nör, 2006; d’Aquino et al., 2007; Carinci et al., 2008; Armiñán et al., 2009; Yao et al., 2008; Wang et al., 2010). DPSCs, Dental pulp stem cells; DFSCs, Dental follicle stem cells; SHEDs, Stem cells populations from human exfoliated deciduous teeth.

In the experiment by Alvarez et al. (Ruth et al., 2015), primary dental pulp cells (DPCs) from the dental pulp tissue of adult third molars were obtained in α-modified Eagle’s medium (containing 20% fetal bovine serum (FBS), non-essential amino acids, 100 units/mL of penicillin, and 100 units/mL of streptomycin) in a humidified incubator at 37 °C with 5% CO2 to obtain DMSCs. Then, fluorescence-activated cell sorting (FACS) technology was used to sort out suitable DMSCs. These cells are induced to undergo odontogenesis through a specific tooth-inducing medium or what is known as odontogenic induction medium (OIM). The results (Table 2) showed that various surface markers (including CD51/CD140α, CD271, and STRO-1/CD146 DMSCs) could be isolated from the surface of DMSCs. Their alkaline phosphatase (ALP) activity was measured to determine their differentiation ability, and the mRNA expression of dental source genes at different time points was detected to confirm that DMSCs with surface markers may exhibit the ability to differentiate into dental lineage cells. Furthermore, numerous researchers have uncovered the differentiation potential of DMSCs. Figure 3 illustrates some of the studies on DMSCs - derived differentiated cells.

**TABLE 2:** ALP activity and expression of dental source marker genes under different induction media cultivation (Ruth et al., 2015).

Experimental group category	Alkaline phosphatase (ALP) activity	The mRNA expression of odontogenic marker genes (including DLX5, RUNX2, BGLAP, DMP1 and DSPP) at different time points
Non-induced CD51/CD140α DMSCs	Control group	/
Induced CD271 DMSCs	Increased by five times compared with the control group	On Day 10, concurrently with the notable induction of BGLAP expression, the expressions of DMP1 and DSPP were significantly upregulated during the induction process
Induced CD51/CD140α DMSCs	Increased by 2.5 times compared with the control group	On Day 7, the expression of DLX5 was significantly increased. On Day 10, the expressions of DMP1 and DSPP were significantly upregulated after induction
Induced STRO-1/CD146 DMSCs	Increased by 1.25 times compared with the control group	On Day 10, the expressions of DMP1 and DSPP were significantly upregulated

### 4.2 Improve the local regenerative microenvironment through immune regulation function

After various injury factors cause damage to the body’s tissues and cells, sentinel cells around the injured tissue (such as macrophages) mediate the production of inflammatory mediators by recognizing injury factors and necrotic tissue. This is the reason why the body often experiences an inflammatory response in cases of tissue damage and infection (such as fractures, trauma, tumors, etc.). The body can repair damage by confining or eliminating injury factors. However, if tissue cells are chronically exposed to an inflammatory environment (such as without using drugs to block the continuous inflammatory process), the inflammatory mediators in the inflammatory environment will activate the host’s vascular response and leukocyte response. The aggregation of a large number of activated cells and the excessive release of enzymes and chemokines will instead impede the process of tissue repair. Timely blocking the development of the inflammatory response through immune regulation function becomes an important factor for tissue regeneration (Wang et al., 2020).

DMSCs can exert immunomodulatory functions by releasing or inhibiting the expression of factors, influencing the proliferation, differentiation, apoptosis and other physiological processes and morphological functions of T cells and macrophages, thereby improving the local regenerative microenvironment to promote tissue repair and regeneration (Table 3).

**TABLE 3:** Immune regulation pathways of DMSCs.

DMSCs	Research scholars	Mechanism of action	Experimental conditions	Specific approaches	References
DPSCs	KWACK et al.	Inhibit the proliferation of T cells	Phytohemagglutinin-activated monocytes, IFN-γ pretreated culture medium	IFN-γ activation of DPSCs can inhibit the proliferation of T cells and reduce the production of IL-17	Kwack et al. (2017)
	ZHAO et al.	Inducing apoptosis of T cells	CD3^+^ T cells activated by phytohemagglutinin	It can inhibit T cell proliferation, induce T cell apoptosis, and stimulate the formation of Treg	Zhao et al. (2012)
	OMI et al.	Influence macrophage polarization	Transplantation of DPSCs into the unilateral hind limb skeletal muscle and DPSCs treated with lipoteichoic acid	Trigger M2 macrophage polarization and inhibit inflammation	Omi et al. (2016)
	RUFAS et al.	Activate the complement system	/	It can express the vast majority of factors required to activate the complement system	Rufas et al. (2016)
PDLSCs	LIU et al.	Inhibit the differentiation of T cells	Periodontal ligament stem cells isolated from an inflammatory environment	Inhibit the secretion of IFN-γ to suppress the differentiation of T cells into Th1	Liu et al. (2012)
	LI et al. SHIN et al.	Inhibit T cell proliferation	Concanavalin A-activated peripheral blood mononuclear cells	Indirect soluble mediators and direct cell-to-cell contact inhibit T cell proliferation, or inhibit the expression of non-classical MHC-like glycoprotein CD1b in DCs to suppress T cell proliferation	Li et al. (2014) Shin et al. (2017)
SHEDs	YAMAZA et al.	Inhibit T cell differentiation	Peripheral blood mononuclear cells activated by anti-CD3/CD28 antibodies and immature CD4^+^	Inhibiting Th17 differentiation, its effect is greater than that of bone marrow mesenchymal cells	Yamaza et al. (2010)
	GAO et al.	Enhance the ability of DC to induce Treg	/	Reduce the secretion of IL-2, TNF-α and IFN-γ by DC and increase the secretion of IL-10	Gao et al. (2018)

DMSCs, Dental mesenchymal stem cells; DPSCs, Dental pulp stem cells; PDLSCs, Periodontal ligament stem cells; SHEDs, Stem cells populations from human exfoliated deciduous teeth.

### 4.3 By releasing a variety of bioactive components, it promotes tissue regeneration

Dental pulp stem cells share the same characteristics as other mesenchymal stem cells, such as high proliferation capacity and multi-lineage differentiation ability. Besides differentiating into various mesodermal tissue cells, dental pulp stem cells also exhibit differentiation capabilities in non-mesodermal tissues, such as neurons. In experiments, researchers have found that dental pulp stem cells in the culture medium for regeneration secrete various bioactive substances. Ogata et al. discovered that the culture medium derived from dental pulp stem cells contains anti-inflammatory cytokines, IL-10, IL-13, follistatin, TGF-β1, hepatocyte growth factor (HGF), neural cell adhesion molecule-1 (NCAM-1), and other bioactive substances. The presence of these bioactive substances is highly beneficial in reducing inflammation, effectively reducing infection, promoting stem cell proliferation, and improving the tissue repair environment (Kwack et al., 2017).

In addition, Zhang et al. found that DPSCs can secrete neuron-related proteins such as DCX, NeuN and NF200, thereby restoring the neurological function of rats with Alzheimer’s disease (Zhang et al., 2021).

Moreover, Roato et al. demonstrated that both PDLSCs and DPSCs can secrete VEGF and generate substances such as proteoglycans when cultured in osteogenic medium (OM). These secretions play a significant role in promoting angiogenesis (Roato et al., 2025).

Through the secretion of growth factors, chemokines, etc., DMSCs exert a paracrine effect. This effect serves to regulate the microenvironment of the injured area, inhibit cell apoptosis and local inflammatory responses, and facilitate angiogenesis and tissue repair.

## 5 The application of DMSCs in the repair of maxillofacial bone tissue

In bone tissue repair, DMSCs can promote bone regeneration, enhance bone repair capacity, improve bone quality, and repair bone defects. They can be applied in periodontal disease treatment and alveolar bone repair. The following is a detailed description of the currently well-studied DPSCs in bone tissue repair.

### 5.1 DPSCs

According to the studies by Amiryaghoubi et al. (2020) and Imanishi et al. (2021), DPSCs were investigated using different materials and scaffolds. The results demonstrated that DPSCs exhibited excellent regenerative performance when supported by hydrogel scaffolds and a mixed scaffold composed of three substances including collagen (COL). This can achieve the goal of micro-repairing mandibular and facial bone defects by efficiently inducing the proliferation of DPSCs and promoting their differentiation into osteoblasts, without inducing inflammation. It is a material that can balance safety and efficiency in mandibular and facial bone regeneration. Meanwhile, Alksne et al. (2022) delved into the biological characteristics of DPSCs themselves and discovered that they can secrete a unique extracellular matrix (ECM) protein network, broadening the understanding of the repair mechanism of DPSCs: this protein network can promote biological behaviors of cells such as adhesion, migration, proliferation or immune regulation by forming a suitable microenvironment, thereby achieving biological processes such as recruiting endogenous stem cells, osteogenesis and angiogenesis (Table 4).

**TABLE 4:** Applications of DPSCs in bone tissue repair.

Cell type	Material/scaffold	Experimental model	Experimental subject	Results and conclusions	References
DPSCs	Injectable thermosensitive hybrid hydrogel containing graphene oxide and chitosan	hydrogel	culture medium	It can significantly upregulate the expression of Runx2 and OCN, enhance ALP activity and mineral deposition	Amiryaghoubi et al. (2020)
DPSCs	The combination of DPSC-EVs and three scaffold materials: COL, β-TCP, and HA.	Skull defect	rat	Micro-CT analysis at 4 weeks and histological examination at 16 weeks revealed that the amount of central bone formation was comparable to that in the live cell transplantation group, and no signs of inflammation were detected	Imanishi et al. (2021)
DPSCs	ECM protein network	Skull defect	rat	It can enhance the ability of cell adhesion, migration and proliferation, and promote osteogenesis and angiogenesis	Alksne et al. (2022)

DPSCs, Dental pulp stem cells; OCN, osteocalcin; ALP, alkaline phosphatase; EVs, Extracellular vesicles; COL, collagen; β-TCP, β- Tricalcium phosphate; HA, hydroxyapatite; ECM, extracellular matrix.

### 5.2 SHEDs

Hiraki et al. (2020) used a 5 mm critical-sized mouse calvarial defect model to demonstrate that both human exfoliated SHEDs and their conditioned medium (SHED-CM) could significantly promote bone regeneration, with SHED-CM showing a superior effect compared to direct SHEDs application. This study was the first to prove that transplanting only the secretome of SHEDs could achieve bone regeneration comparable to that of live cells without immunosuppression, making it particularly suitable for repairing alveolar clefts in children with cleft lip and palate. Sattary et al. (2022) used polycaprolactone/gelatin/hydroxyapatite nanoparticles (PGH) as a scaffold to culture SHEDs and found that PGH scaffolds seeded with SHEDs could accelerate bone repair, which could be further translated into clinical applications for repairing craniofacial bone defects. Kunwong et al. (2021) further explored the application of SHEDs in a poly (lactic-co-glycolic acid) (PLGA)-bioactive glass composite scaffold. The results showed that a scaffold composed of 10% bioactive glass and PLGA performed well in promoting the adhesion, proliferation, and osteogenic differentiation of SHEDs, and could promote new bone formation at the site of rat alveolar bone defects without inducing inflammatory responses, providing important experimental evidence for the development of new and efficient bone regeneration materials (Table 5).

**TABLE 5:** Applications of SHEDs in bone tissue repair.

Cell type	Material/scaffold	Experimental model	Experimental subject	Results and conclusions	References
SHEDs	SHED-CM	Skull defect	mouse	It can significantly increase the volume of new bone, the proportion of mature bone, and vascular density, and is rich in osteogenic and angiogenic factors	Hiraki et al. (2020)
SHEDs	PGH	Skull defect	mouse	The expression of osteogenic genes such as ALP, RUNX2, and OCN was significantly upregulated. The amount of new bone formed within 8 weeks was high, and mature bone and blood vessels grew in synchrony without any inflammatory response	Sattary et al. (2022)
SHEDs	PLGA-10% bioactive glass composite scaffold	Alveolar bone cleft	rat	Within 14 days, the activity of ALP and the expression of RUNX2, OCN and Col1 were significantly increased, mature bone matrix could be formed, and osteocalcin was positive	Kunwong et al. (2021)

SHEDs, Stem cells populations from human exfoliated deciduous teeth; CM, conditioned medium; PGH, Polycaprolactone/gelatin/hydroxyapatite Nanoparticles; PLGA, Poly (lactic-co-glycolic acid); ALP, alkaline phosphatase; OCN, osteocalcin.

### 5.3 SCAPs

Cui et al. (2020) demonstrated that injecting berberine (BBR) into the root canals of a rat model of apical periodontitis activated the Wnt/β-catenin signaling pathway in SCAPs, significantly promoting root apex repair in immature teeth with apical periodontitis. This finding suggests that pharmacological activation of SCAPs can drive root reconstruction and may be used for the treatment of root apex defects in children and adolescents. Wu L. et al. (2021) found that a low concentration (0.2 mg/mL) of injectable root canal sealer (iRoot SP) significantly promoted the proliferation, migration, and osteogenic differentiation of SCAPs. By regulating the activity of SCAPs, it provides a favorable microenvironment for the repair of periapical bone tissue and is expected to become an efficient biomaterial for promoting periapical bone regeneration. Ramírez et al. (2024) discovered that both platelet-rich fibrin (PRF) and low-level laser therapy (LLLT) alone could promote the osteogenic differentiation of SCAPs, and their combined use produced a synergistic effect, significantly enhancing the formation of mineralized nodules and the secretion of osteogenic-related proteins (such as BMP-2 and BMP-4) by SCAPs. This study indicates that the combination of PRF and LLLT can serve as an efficient tool for the repair of maxillofacial bone tissue, providing a new combined treatment strategy and holding promise for more effective bone regeneration in clinical practice (Table 6).

**TABLE 6:** Applications of SCAPs in bone tissue repair.

Cell type	Material/scaffold	Experimental model	Experimental subject	Results and conclusions	References
SCAPs	BBR	Immature teeth with apical periodontitis	rat	μCT results at 3 weeks indicated remarkable root reconstruction. Significantly enhanced expression levels of Runx2, ALP, and Col1α1 were observed, which effectively promoted the formation of mineralized nodules	Cui et al. (2020)
SCAPs	0.2 mg/mL iRoot SP	Osteogenic culture medium	culture medium	It can enhance ALP activity and the ability to form mineralized nodules, significantly up-regulating the mRNA and protein expressions of OCN, OSX, Runx2 and DSPP.	Wu L. et al. (2021)
SCAPs	LLLT combined with platelet-rich fibrin (PRF)	Osteogenic culture medium	culture medium	It can promote the formation of osteogenic nodules, significantly upregulate the expression of genes such as OCN, OPN, MSX1, TGF-β1 and SMAD1, as well as the protein levels of BMP-2 and BMP-4	Ramírez et al. (2024)

SCAPs, Stem cells from apical papilla; BBR, berberin; ALP, alkaline phosphatase; iRoot SP, injectable root canal sealer; LLLT, Low-level laser therapy; PRF, Platelet-rich fibrin; OCN, osteocalcin; OPN, osteopontin.

### 5.4 PDLSCs

Pan et al. (2020) encapsulated PDLSCs in liquid methacrylated Gelatin (GelMA) hydrogel and demonstrated in a rat critical alveolar bone defect model that GelMA hydrogel could effectively fill any irregularly shaped alveolar bone defects and provide a suitable physicochemical microenvironment for the adhesion, viability, and osteogenic differentiation of PDLSCs, protecting PDLSCs from being lost due to bone defects in the early stage of transplantation. PDLSCs encapsulated in GelMA hydrogel exhibited significant alveolar bone regeneration potential. Ha and Choung (2020) proved that methylsulfonylmethane (MSM) could significantly promote the proliferation and osteogenic differentiation of PDLSCs and enhance the regeneration efficiency of PDLSCs in the repair of maxillofacial bone defects. Zhao et al. (2022) demonstrated that exosomes derived from periodontal ligament stem cells (PDLSCs-Exos) could significantly promote the repair of mandibular bone defects. Their combined application with sodium GelMA-Alginate hydrogel (Gel-Alg) could be used to repair initial alveolar bone defects in SD rats, promoting bone regeneration. This is a promising strategy for the repair of mandibular bone defects, breaking through the limitations of traditional stem cell therapy and providing new ideas for future clinical applications (Table 7).

**TABLE 7:** Applications of PDLSCs in bone tissue repair.

Cell type	Material/scaffold	Experimental model	Experimental subject	Results and conclusions	References
PDLSCs	GelMA hydrogel	Alveolar bone defect	rat	The bone volume fraction was as high as 67.2% at 8 weeks, with abundant new bone and high ALP activity	Pan et al. (2020)
PDLSCs	MSM	Skull defect	mouse	The expression of genes and proteins such as ALP, OPN, OCN, and Runx2 doubles, which can promote osteogenic differentiation *in vitro* and *in vivo* and produce bone matrix	Ha and Choung (2020)
PDLSCs	The combination of PDLSCs-Exos and Gel-Alg hydrogel	Alveolar bone defect	rat	It significantly promotes the proliferation and osteogenic differentiation of bone marrow mesenchymal stem cells, enhances their ALP activity, and can form more new bone	Zhao et al. (2022)

PDLSCs, Periodontal ligament stem cells; GelMA, methacrylated gelatin; ALP, alkaline phosphatase; OPN, osteopontin; OCN, osteocalcin; MSM, methylsulfonylmethane; Exos, Exosomes; Gel-Alg, GelMA-Alginate.

### 5.5 DFSCs

Meng et al. (2022) focused on the effects of N-acetylcysteine (NAC) on the osteogenic properties of DFSCs and alveolar bone repair. The study found that treatment with 5 mM NAC significantly enhanced the proliferation ability of DFSCs, reduced the senescence rate, and increased the expression of stem cell-specific markers and immune-related factors. It also promoted the strongest osteogenic differentiation ability. An appropriate concentration of NAC enhanced the osteogenic properties of DFSCs through the PI3K/AKT/ROS signaling pathway, providing clinical potential for alveolar bone regeneration based on stem cells. Chen et al. (2023) revealed the important role of long non-coding RNA HOTAIRM1 in DFSC-mediated bone regeneration. The study found that HOTAIRM1 was induced under hypoxic conditions and activated HIF-1α, which in turn upregulated oxygen-sensing histone demethylases KDM6A/B and inhibited the methyltransferase EZH2. By targeting HIF-1α, HOTAIRM1 regulated the demethylation of H3K27 and promoted osteogenic differentiation of DFSCs. This study provided a new molecular mechanism and potential therapeutic target for the application of DFSCs in the repair of maxillofacial bone tissue, indicating that HOTAIRM1 may promote the osteogenic ability of DFSCs and bone regeneration by regulating the HIF-1α/KDM6/EZH2/H3K27me3 axis (Table 8).

**TABLE 8:** Application of DFSCs in bone tissue repair.

Cell type	Material/scaffold	Experimental model	Experimental subject	Results and conclusions	References
DFSCs	NAC	Alveolar bone defect	rat	It has the potential to enhance the osteogenic differentiation ability. It enriches genes associated with the “developmental process”, decreases the intracellular ROS levels, and strengthens the antioxidant capacity	Meng et al. (2022)
DFSCs	HOTAIRM1	Skull defect	rat	Regulating the demethylation of H3K27 promotes osteogenic differentiation of DFSCs and shows stronger ALP activity and mineralized nodule formation ability under hypoxic conditions	Chen et al. (2023)

DFSCs, Dental follicle stem cells; NAC, N-acetylcysteine; ROS, reactive oxygen species; ALP, alkaline phosphatase.

## 6 The application of DMSCs in the repair of soft tissues in the maxillofacial region

The oral and maxillofacial region plays a crucial role in chewing, pronunciation, and facial expressions. However, its high exposure rate makes it vulnerable to trauma and infection. Defects in the soft tissues of the oral and maxillofacial region not only cause functional problems for patients, bringing many inconveniences to their lives, but also seriously affect their appearance and mental health. In recent years, as people’s demands for aesthetic restoration have increased, how to functionally reconstruct the damaged tissues while restoring their appearance as much as possible and achieving good aesthetic restoration results has become the focus of soft tissue repair. Utilizing dental mesenchymal stem cells to achieve soft tissue regeneration in the oral and maxillofacial region will become an effective approach for treating and restoring maxillofacial soft tissues in the future.

### 6.1 PDLSCs

The periodontal ligament is a connective tissue located between the alveolar bone and cementum, capable of absorbing mechanical pressure exerted on the teeth during chewing. PDLSCs are heterogeneous, clonal, highly proliferative and pluripotent cells that can differentiate into osteoblasts, cementoblasts, chondrocytes and adipocytes, promoting the new formation of periodontal tissues, including periodontal ligament fibers similar to Sharpey fibers, bone and cementum (Queiroz et al., 2021).

You et al. (2024) confirmed through a rat periodontal window defect model that PDLSCs injection could reduce the reverse diversity during periodontal repair and increase the abundance of non-pathogenic bacteria such as Bifidobacterium and *Lactobacillus*, demonstrating that PDLSCs have the ability to influence the oral microbiota and promote periodontal regeneration. Meanwhile, the studies by Zhao et al. (2020) and Hu et al. (2020) indicated that PDLSCs, when combined with different carriers, could improve periodontal tissue regeneration. They found that the expression of type I collagen, alkaline phosphatase and bone sialoprotein genes was significantly upregulated on the polycaprolactone (PCL) membrane scaffold containing simvastatin (SIM), and the formation of mineralized matrix increased. In the tissue derived from PDLSC sheets combined with HA/TCP, the fibers showed obvious directionality, meeting the functional requirements of the periodontal soft tissue for the ordered structure of fibers. This suggests that PDLSCs materials provide a molecular and structural basis for the repair of periodontal soft tissue, which may contribute to periodontal tissue regeneration (Table 9).

**TABLE 9:** Research on PDLSCs in soft tissue repair.

Author	Year	Subjects	Source of stem cells	Carrier	Results and conclusions	References
You et al.	2024	rat	human	/	Injection of PDLSCs can enhance periodontal regeneration and regulate oral microbiota	You et al. (2024)
Zhao et al.	2020	Athymic nude mice	human	PCL-SIM	Promoting the formation of cementum-like tissue, it has the feasibility of improving periodontal regeneration	Zhao et al. (2020)
Hu et al.	2020	Athymic nude mice	human	HA/TCP	PDLSCs have the ability to form mineralized tissue and express proteins related to periodontal ligament tissue	Hu et al. (2020)

PDLSCs, Periodontal ligament stem cells; PCL, polycaprolactone; SIM, simvastatin; HA, hydroxyapatite; TCP, tricalcium phosphate.

### 6.2 SCAPs

SCAPs are a type of MSC population residing in the apical region of immature permanent teeth, characterized by high proliferation potential, self-renewal ability, and low immunogenicity (Sonoyama et al., 2006), and thus hold great potential for stem cell therapy.

Combining the *in vitro* and *in vivo* experimental results of Jing et al. (2022) on the exosomes of stem cells from apical papillae (SCAP-Exo), it was found that SCAP-Exo demonstrated excellent angiogenic and osteogenic properties, and this ability was still significantly manifested in diabetic (diabetes mellitus, DM) rats. The experiments showed an increase in the number of trabeculae in the alveolar bone of DM rats, a reduction in trabecular space, and a significant improvement in bone healing. This indicates that SCAPs have outstanding potential in promoting periodontal tissue regeneration. Meanwhile, Li et al. (2018) focused on the clinical translation potential of SCAPs, taking the key clinical assessment indicators in periodontitis treatment - probing pocket depth (PPD) and attachment loss (AL) - as the core research objects. Through experiments, it was confirmed that SCAPs could effectively improve the PPD and AL indicators in a porcine periodontitis model. By using clinical assessment data such as PPD and AL, a bridge was built between laboratory research and clinical application, which has a positive effect on the application of SCAPs in soft tissue repair (Table 10).

**TABLE 10:** Research on SCAPs in soft tissue repair.

Author	Year	Subjects	Source of stem cells	Carrier	Results and conclusions	References
Jing et al.	2022	Diabetic rats	human	PEG/DNA hybrid hydrogel	SCAP-Exo can promote angiogenesis and bone regeneration	Jing et al. (2022)
Li et al.	2018	Porcine periodontitis model	human	/	The improvement of the gingival condition promoted the regeneration of periodontal tissues	Li et al. (2018)

SCAPs, Stem cells from apical papilla; PEG, polyethylene glycol; Exo, exosome.

### 6.3 SHEDs

SHEDs are regarded as a promising cell population for cell-based or cell-free therapy and tissue engineering due to their proliferative, pluripotent and immunomodulatory properties. Studies have found that in addition to their osteogenic and odontogenic potential, SHEDs also exhibit remarkable neuroregenerative capabilities (Tsutsui, 2020).

According to the research of Wu M. et al. (2021), it was found that the exosomes of SHEDs (SA-Exo) can upregulate the expression of angiogenesis-related proteins such as VEGF, angiopoietin 2 and PDGF in SHEDs. This reflects the endothelial differentiation potential of SHEDs and also indicates from a histological perspective that SA-Exo can enhance the regeneration of the dentin-pulp complex and angiogenesis. Guo et al. (2020) focused on the regeneration of blood vessels and nerves. Through histological analysis, it was shown that the extracted pulp permanent incisors of miniature pigs implanted with SHEDs contained regenerated pulp tissue with a similar structure to normal pulp. Moreover, the positive expression of platelet endothelial cell adhesion molecule (CD31) and neurofilament (NF) in the regenerated pulp tissue confirmed the regeneration of blood vessels and nerves. In summary, SHEDs have the ability to regenerate host pulp tissue *in vivo*. The clinical case report by Ghana Shyam Prasad et al. (2019) provided key practical evidence: after treating tooth #46 with SHEDs for apical periodontal lesions, the patient’s periapical radiolucency completely disappeared, and tooth #46 responded positively to the electric pulp test, which may indicate the reinnervation of pulp tissue in the root canal. It can be seen that the application of SHEDs in humans shows the ability to effectively treat apical periodontal lesions of permanent teeth (Table 11).

**TABLE 11:** Research on SHEDs in soft tissue repair.

Author	Year	Subjects	Source of stem cells	Results and conclusions	References
Wu et al.	2021	Immune-deficient mouse model	deciduous teeth	It can promote the regeneration of the dentin-pulp complex	Wu M. et al. (2021)
Guo et al.	2020	Porcine dental pulp regeneration model	deciduous teeth	The accompanying blood vessels and nerves were discovered, suggesting that the regenerated dental pulp may be capable of sensing external stimuli	Guo et al. (2020)
Ghana et al.	2019	A 12-year-old patient with apical periodontitis	The same child’s deciduous teeth	Four months after the operation, regenerated dental pulp tissue appeared in the root canal	Ghana Shyam Prasad et al. (2019)

SHEDs, Stem cells populations from human exfoliated deciduous teeth.

### 6.4 GMSCs

The gingiva is firmly attached to the teeth and jawbone, forming a mucoperiosteum, which has the ability to resist masticatory and minor traumatic forces. It plays a crucial role in maintaining oral health and exhibits a unique fetal-like scarless healing process after injury, suggesting that this tissue may be a source of stem cells (Chen et al., 2012).

Qiu et al. (2020) transplanted GMSCs into periodontal defects in rats and found that periodontal tissue regeneration occurred, with significantly lower expression levels of tumor necrosis factor (TNF-α) and interleukin (IL-1β) compared to the control group, and significantly higher expression of BSP-II and Runx2. This indicates that GMSC-CM may have the effect of inhibiting inflammation, promoting periodontal regeneration, and osteogenic differentiation. Sun et al. (2019) transplanted GMSCs into C57BL/6 J mice with periodontitis and found that the alveolar bone height of the mice increased significantly, and GMSCs were detected in the newly formed periodontal ligament and alveolar bone. This suggests that systemically transplanted GMSCs can return to the periodontal injury site and promote the regeneration and repair of periodontal tissue. Fawzy El-Sayed et al. (2015) transplanted GMSCs into periodontal defects in miniature pigs and found that compared to the control group, they showed better clinical attachment levels, PPD, GR, periodontal attachment levels, cementum regeneration and bone regeneration, as well as shorter junctional epithelium length and better improvement in probing bleeding. This indicates that GMSCs have excellent potential for periodontal regeneration (Table 12).

**TABLE 12:** Research on GMSCs in soft tissue repair.

Author	Year	Subjects	Source of stem cells	Results and conclusions	References
Qiu et al.	2020	Rat periodontal defect model	human	GMSCs transplantation promoted periodontal defect regeneration in rats, inhibited inflammation and promoted osteogenic differentiation	Qiu et al. (2020)
Sun et al.	2019	Mouse periodontitis model	human	GMSCs can locate the sites of inflamed periodontal tissues and be used for the formation of new tissues	Sun et al. (2019)
Fawzy et al.	2015	Miniature pig periodontitis model	Self	It demonstrated the significant periodontal regeneration potential of GMSCs	Fawzy El-Sayed et al. (2015)

GMSCs, Gingival mesenchymal stem cells.

## 7 Summary and prospects

In summary, DMSCs have demonstrated excellent performance in preclinical and clinical studies for the repair of bone and cartilage tissues in the maxillofacial region under the support of various culture media. Despite the promising application prospects of DMSCs, there are still bottlenecks for their large-scale clinical application.

### 7.1 Challenges in the clinical application of DMSCs

#### 7.1.1 Lack of standardized isolation and culture procedures for DMSCs

One of the challenges in the clinical application of DMSCs is how to obtain a large number of stem cells from tissues. This goal cannot be achieved without the isolation of stem cells, multiple passages, and cryopreservation. Although enzymatic methods are generally applicable in the process of isolating stem cells, there are no unified regulations for the physical operations of tissue separation for different types of DMSCs. Moreover, multiple passages may reduce the differentiation potential of cultured cells, thus leading to the development of various culture media to regulate their performance. Additionally, different cryopreservation strategies, such as controlled-rate freezing and non-controlled-rate freezing, are still controversial (Khaseb et al., 2021).

#### 7.1.2 Difficulties in quality control of DMSCs

Compared with chemically synthesized drugs, the cell culture conditions of DMSCs, including cell passage number and density, may lead to inconsistencies among different batches of DMSCs. Moreover, studies have found that the conditioned medium functions of DMSCs from different sources may be affected by their cell sources (Joo et al., 2018). The age and diseases of DMSC donors may also influence the composition of DMSC culture media (Bhandi et al., 2021).

#### 7.1.3 Rejection and tumorigenic risks

When DMSCs are used for allogeneic transplantation, the risks of immunogenicity and tumorigenicity cannot be ignored. Since DMSCs, such as DPSCs, have unique immunomodulatory functions, if their upregulated genes are enriched in immune and inflammatory-related cell pathways, they often exhibit higher immunogenicity, increasing the risk of immune rejection during allogeneic transplantation. Additionally, the differential expression of tumor-related genes and the presence of undifferentiated induced pluripotent stem cells during the induction and differentiation process of DMSCs may also lead to tumor formation (Taoran et al., 2024).

#### 7.1.4 Cost issues in large-scale production

Exosomes are extracellular vesicles that carry various bioactive molecules such as proteins, genetic material, and lipids, playing a crucial role in intercellular communication and material transport (Lu et al., 2024). Exosomes can be extracted from DMSCs, and studies have found that their functions are highly dependent on the source cells and their physiological states (Huang et al., 2016). In recent years, multiple studies have discovered that exosomes play a role in various aspects such as angiogenesis (Ribeiro et al., 2013) and neural repair (Jarmalavičiūtė et al., 2015) by regulating intracellular signaling pathways. Like other bioactive components, exosomes, as an important component of DMSCs, must be stored under appropriate conditions. Wu J et al. (2021) found that different exosome storage conditions can affect their biological utilization rate. Generally, it is recommended to store them at −80 °C, but this condition is costly (Zhang et al., 2025).

Although a large number of proteins, nucleic acids, lipids and metabolic enzymes have been identified in Exos, little is known about their functions and sorting mechanisms. Moreover, the concentration of effective substances in Exos depends on the surrounding environment and the metabolic state of the host cells, making the condition control in the clinical application of Exos rather complex. Despite extensive research on Exos in chronic wound healing and skin regeneration, the exact molecular mechanisms and roles of Exos in tissue repair and regeneration still need further investigation. In addition, to use Exos as a research tool, extensive research is still needed in the fields of EV biosynthesis, cellular uptake and transport, which still has a long way to go (Hade et al., 2022).

### 7.2 Prospects for the clinical application of DMSCs

DMSCs, with their multi-directional differentiation and immunomodulatory properties, show great potential in the repair of maxillofacial defects. In the future, through technological optimization and more preclinical and clinical trials, overcoming issues such as quality control and safety, DMSCs may become a core force in regenerative medicine.

## Glossary

DMSCs: Dental mesenchymal stem cells
MSCs: Mesenchymal stem cells
DPSCs: Dental pulp stem cells
PDLSCs: Periodontal ligament stem cells
SHEDs: Stem cells populations from human exfoliated deciduous teeth
GMSCs: Gingival mesenchymal stem cells
DFSCs: Dental follicle stem cells
DFPCs: Dental follicle progenitor cells
ABMSCs: Alveolar bone mesenchymal stem cells
SCAPs: Stem cells from apical papilla
DPCs: Dental pulp cells
FBS: Fetal bovine serum
FACS: Fluorescence-activated cell sorting
OIM: Odontogenic induction medium
ALP: Alkaline phosphatase
HGF: Hepatocyte growth factor
NCAM-1: Neural cell adhesion molecule 1
OM: Osteogenic medium
EV: Extracellular vesicles
COL: Collagen
β-TCP: β-Tricalcium phosphate
HA: Hydroxyapatite
ECM: Extracellular matrix
CM: Conditioned medium
PGH: Polycaprolactone/gelatin/hydroxyapatite Nanoparticles
PLGA: Poly(lactic-co-glycolic acid)
BBR: Berberin
iRoot SP: Injectable root canal sealer
PRF: Platelet-rich fibrin
LLLT: Low-level laser therapy
OCN: Osteocalcin
OPN: Osteopontin
**GelMA**-: Methacrylated Gelatin
MSM: Methylsulfonylmethane
Exos: Exosomes
Gel-Alg: GelMA-Alginate
BMSCs: Bone marrow mesenchymal stem cells
NAC: N-acetylcysteine
ROS: Reactive oxygen species
SIM: Simvastatin
PCL: Polycaprolactone
DM: Diabetes mellitus
PEG: Polyethylene glycol
PPD: Probing pocket depth
GR: Gingival recession
AL: Adhesion loss
PDGF: Platelet-derived growth factor
NF: Neurofilament
CCM: Concentrated conditioned medium
